# Development and bioassessment of high nutria-omega 5 cookies through animal modeling

**DOI:** 10.3389/fnut.2023.1199645

**Published:** 2023-06-30

**Authors:** Nida Iqbal, Muhammad Asim Shabbir, Moazzam Rafiq Khan, Muhammad Naeem Faisal

**Affiliations:** ^1^Faculty of Food Nutrition and Home Sciences, National Institute of Food Science and Technology, University of Agriculture, Faisalabad, Pakistan; ^2^Faculty of Veterinary Sciences, Institute of Physiology and Pharmacology, University of Agriculture, Faisalabad, Pakistan

**Keywords:** agro-industrial waste, malnutrition, high nutria-omega 5 cookies, sunflower meal protein concentrate, pomegranate seed oil

## Abstract

The food industry generates a diverse range of waste byproducts during fruit processing, which can be repurposed to create functional foods and other valuable commodities. In this particular study, leftover agro-waste from pomegranate juice was valorized to obtain pomegranate seed oil (PSO), while utilizing sunflower oilseed cake to produce sunflower meal protein concentrate (SMPC). These two extracted components were then combined as ingredients to produce High Nutria Omega 5 (HNO5) cookies. To ensure the quality and viability of pomegranate seed oil, a comprehensive set of laboratory analytical procedures were employed to evaluate its characteristics. Subsequently, different ratios of pomegranate seed oil and sunflower meal protein concentrate were utilized to develop the HNO5 cookie products. These cookies underwent thorough sensory, physicochemical, storage, and proximate evaluations as well as efficacy studies to assess their overall nutritional quality and shelf-life properties. As compared to the control feed, the findings of the renal and liver functional tests indicated a favorable effect on ALT, AST, ALP, serum urea, creatinine, albumin, globulins, total proteins, and A/G ratio. The results revealed that PSO and SMPC cookies containing 15% PSO and 15% SMPC exhibited stability in numerous physicochemical and sensory assessments. The punicic acid in HNO5 cookies significantly reduced the effects of starvation in rats and progressively improved several metabolic processes and overall health profiles.

**Graphical Abstract:**
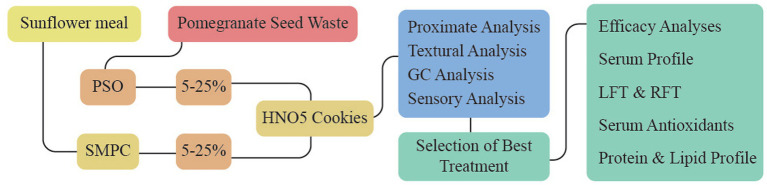

## 1. Introduction

The pomegranate is one of the oldest fruit plants named “*Punica granatum*,” which means “seeded apple” or “grainy apple,” driven from the word *Ponum granatum* that has been cited in the Ancient Egyptian documents and the Bible. It is also considered an appealing, pleasant and nutritious fruit due to its rich nutritional properties (1). Pomegranate fruit and its components, such as seed, peel and juice, have a higher antioxidant capacity and other valuable features, motivating scientists to find more latent and beneficial bioactive chemicals for food applications and nutraceuticals (2).

Sunflowers are short-season plants belonging to the genus Helianthus and Asteraceae family, and there are more than seventy classes all over the globe. It originated in the temperate parts of North America (20 to 25°C) and was known to European travelers in the sixteenth century (3). Some essential amino acids have also been found in sunflower products, including histidine, threonine, glutamic acid, valine, tyrosine, arginine, methionine, serine, cysteine, alanine, lysine, isoleucine, aspartic acid, glycine phenylalanine and proline (4). Pomegranate has a similar property to *Portulaca oleracea* (purslane), and *Abelmoschus esculentus* (okra) which have wide range of pharmacological effects, including antibacterial, analgesic, anti-inflammatory, and wound-healing qualities. Okra, a member of the Malvaceae family, supplies vital fatty acids for human nutrition in its seed oil. Numerous studies have suggested that okra seed oil can decrease cholesterol. Okra seeds are used to create 20–40% of the world’s oil (5, 6).

De-oiled sunflower cake (DC) is a promising and inexpensive protein source due to its high protein content. A long-term plant-based protein manufacturing approach requires the revaluation of industrial waste. There is no report in the literature regarding any toxic or allergic compounds found in sunflower protein (SP) or any genetic alteration. Because of these features, there has been a recent spike in industrial and scientific interest in sunflower protein, particularly for protein extraction or other human ingesting possibilities, such as flour replenishment in bakery items (7, 8). Cookies are small, sweet baked commodities made with flour, eggs, sugar, butter, cooking oil, or other fat. Three basic ingredients are involved in its preparation, i.e., fat, flour and sugar (9).

Malnutrition refers to deficiency or excess of any nutrient in a person’s energy intake. Malnourished portions lack nutritious and energetic food, notably lacking protein, vitamins and trace minerals. Pakistan is facing malnutrition that disproportionally disturbs almost all females. People belonging to low-income families are more prone to malnutrition and infectious diseases. The prevalence of malnutrition (underweight, stunting, and wasting) increases children’s mortality risk. Vitamin and iron inadequacy are micronutrient-related malnutrition (10).

Considering the facts mentioned earlier, pomegranate seed oil and sunflower meal protein concentrate can be valuable ingredients in ameliorating malnutrition. The functional properties of punicic acid and protein isolates obtained through extraction used in the formulation of High nutria omega 5 (HNO5) cookies were also estimated. For bio-evaluation, tests were performed on experimental rats to determine the influence of the resulting cookies on their suitability, efficiency, and scope of use. The primary goals of this study encompassed the utilization of waste sunflower and pomegranate seeds as valuable resources for preparing value-added ingredients. Additionally, the study aimed to create high nutria-omega 5 cookies with enriched properties of SMPC and PSO while also investigating their storage stability. Furthermore, an efficacy study was conducted to evaluate the potential health benefits of consuming these high nutria-omega 5 cookies.

## 2. Materials and methods

The discarded pomegranate seeds and sunflower meal were obtained from a local seller in Faisalabad, Pakistan.

### 2.1. Oil extraction

The oil was extracted using the Soxhlet extraction technique from the clean and dry powder of pomegranate seeds, as described by Jing et al. (11). The sample was dried and added to a thimble; n-hexane solvent was used for Soxhlet extraction. The obtained pomegranate seed oil (PSO) was purified through rotary evaporation and kept at −20°C in amber-colored bottles for further evaluation.

### 2.2. Fatty acid composition

PSO was converted to fatty acid methyl esters (FAME) using the method described and adopted by Khoddami et al. (12) and Ledoux et al. (13), with some changes. A 50 mg oil sample was trans-esterified with 50 μL sodium methoxide and 950 μL n-hexane. After vortexing for 5 s and settling for 5 min, a 1 μL aliquot of the top layer was taken for Gas Chromatograph (GC) analysis.

### 2.3. Preparation of sunflower meal protein concentrate

Milled sunflower meal was concentrated according to the procedure described by Salgado et al. (14) and Lovatto et al. (15) with some modifications. Sunflower oilseed cake (67 g/L) was mixed with water and stirred for 1 h at pH 9 using NaOH (3 mol/L). The mixture was then separated using a basket type centrifuge at 2100 G (RCF) and 20°C for 30 min. The resulting supernatants from protein extraction were combined and an isoelectric precipitation was conducted by adding 3 mol/L until reaching pH 9.

### 2.4. Crude protein

The crude protein contents of SMPC were estimated according to Kjeldahl’s method as described in AACC (2000) Method No. 46-10.

### 2.5. Amino acid characterization

The amino acid profiling of SMPC was performed with the help of an amino acid analyzer. The sample preparation was done according to the method recommended by EU Directive (16).

### 2.6. Product development

The cookies were prepared according to the procedure described in AACC (17) Method No. 10-50d. According to the formulation of various treatments, cookies were designed using different concentrations of sunflower meal protein concentrate (Supplementary Table S1).

### 2.7. Analysis of high nutria omega 5 cookies

The prepared High Nutria Omega 5 cookies were subjected to various quality analyses, and their storage stability was investigated at different storage intervals (0, 15, 30, 45, and 60 days).

#### 2.7.1. Crude protein

The crude protein content of sunflower protein concentrate samples was determined using the Kjeldahl’s method No. 46-10 is mentioned in AACC (17).

#### 2.7.2. Textural analysis

Following the method described by Chauhan et al. (18), the textural parameter was calculated using a 5 kg load cell texture analyzer (Model TA-XT2, Secure Microsystems and Surrey, UK).

#### 2.7.3. GC analysis

The fatty acid analysis was performed as described in Section 2.2.

#### 2.7.4. Energy value

The energy value of HNO5 cookies was calculated using the Oxygen Bomb Calorimeter (IKA-WERKE, C2000 Basic, GMBH and Co., Germany), as defined by Krishna and Ranjhan (19).

#### 2.7.5. Sensory evaluation of cookies

The taste panel scored the cookies of various treatments using the 9-point hedonic scoring scale (20).

#### 2.7.6. Selection of best treatment of cookies

The best treatment was selected based on storage stability and sensory evaluation for the efficacy study.

### 2.8. Experimental plan

An *in-vivo* study was conducted using 50 eight-week-old Sprague Dawley rats, weighing an average of 280 ± 10 g. The rats were obtained from the National Institute of Food Science and Technology. They were housed in metabolic cages at room temperature (25°C) with a 12:12 light–dark cycle. The study followed the guidelines for animal care and use, approved by the Institutional Biosafety/Bioethics Committee. The rats were acclimated to the environment and provided with a regular diet and water *ad libitum* for three days. For the experiment, the rats were randomly divided into five groups, each consisting of 10 rats (Supplementary Table S2). The ingredients of all experimental diet plans are shown in Supplementary Table S3. The first group served as the reference and received a standard diet throughout the experiment. The other four groups of rats underwent eight days of long-term starvation, resulting in weight loss (average of 190 ± 10 g). After starvation, the G0 group received a basal diet for 45 days, while G1 and G2 were given 15% control and 15% HNO5 (high fat and high protein) cookies incorporated into their feed for the same period. The diets for G3 and G4 were equivalent to the protein and fat ratios found in HNO5 cookies. The experimental diets were compared to the control groups (G0 and G1). The study aimed to assess the specific benefits of PSO, SMPC, and HNO5 cookies by re-feeding the starved rats. Evaluations were conducted at 0, 15, 30, and 45 days after the rats were re-fed following the eight-day starvation period. On the final day, the animals were euthanized for further analysis.

### 2.9. Analysis of serum profile

According to the standard method, the serum from the blood sample was separated using the centrifuged machine for serum analyses.

#### 2.9.1. Level of punicic acid

Punicic acid levels in serum were determined when the lipids were extracted from rat serum using the method Yuan et al. (21) explicated.

#### 2.9.2. GC analysis

The FAMEs were analyzed on a GC column system according to the protocol illustrated by Benner et al. (22) with some modifications. A GC system with a flame ionization detector was utilized, equipped with a biscyanopropyl polysiloxane capillary column (100 m × 0.25 mm × 0.25 μm). The injection port and the detector were kept at 250°C. FAME peaks were identified by comparing their retention time to a reference standard. Punicic acid content was expressed as a percentage of fatty acids in lipid fractions.

#### 2.9.3. Serum proteins

Following the manufacturer’s instructions, commercially available kits (Bioclin® Total Protein Monoreagent Diagnostic Kit; K031 and Bioclin® Albumin Monoreagent Diagnostic Kit; K040) were used to assess total protein and albumin concentrations in serum samples. The serum globulin concentration in each piece was calculated by subtracting the serum albumin concentration from the total protein concentration.

#### 2.9.4. Protein quality evaluation

The protein efficiency ratio (PER) was determined by following the equation developed by Henry (23), which corresponded to the ratio of protein intake and body weight. Similarly, the net protein ratio (NPR) was calculated using body weight loss observed through a protein-free diet in the control cookies group by following the equation described by Bender and Doell (24).

### 2.10. Serum antioxidants

The total antioxidant capacity (TAC) in the serum samples and serum total oxidative stress (TOS) were determined using the novel automated calorimetric approach as described by Erel (25).

#### 2.11. Lipid profile

Serum concentrations of total cholesterol (TC), triglycerides, high-density lipoprotein cholesterol (HDL), and low-density lipoprotein cholesterol (LDL) were measured using the Trinder enzymatic method using Liquiform Cholesterol kit (Lab test Diagnostics, Brazil).

### 2.12. Statistical analysis

The collected data was finalized, and outcomes from contemporary research were statistically analyzed by applying a complete randomized design (CRD) with Tukey’s HSD *post hoc* test for further analysis according to the protocol defined by Montgomery (26).

## 3. Results and discussions

The objective of the present study was to characterize the significance of pomegranate seed oil (PSO) and sunflower meal protein concentrate (SMPC) with regards to nutritional and functional properties of the developed edible product (High nutria Omega-5 (HNO5) cookies) to treat malnutrition related consequences.

### 3.1. Fatty acid composition of pomegranate seed oil

The results regarding the fatty acid profile are shown in Table 1. The major fatty acid found in oil samples was conjugated linolenic acid (CLnA, C18:3), known as punicic acid, one of PSO’s principal and active components responsible for its antioxidant potential (27). Conjugated linolenic acid was found as abundant as 73.21%. The other fatty acids observed included linoleic acid (CLA, C18:2), which was 5.45%, followed by oleic acid (C18:1), 10.24%. However, myristic (C14:0), stearic (C18:0) and palmitic (C16:0) acids were found in lesser extent, i.e., 0.31, 2.65, and 5.12%, respectively. Total amount of saturated fatty acids (SFAs) was 8.29%, whereas unsaturated fatty acids (MUFAs and PUFAs) was 90.12%. The presence of punicic acid is linked to the biological and health benefits of pomegranate seed oil (28). According to Siano et al. (29), punicic acid is stable at 50°C; heating it at 170°C for 4 h causes weak isomerization into positional and geometrical isomers.

**Table 1:** Concentrations for fatty acid composition in PSO (Wt. %).

PSO	Concentration
Myristic acid (C14:0)	0.31
Palmitic acid (C16:0)	5.12
Stearic acid (C18:0)	2.65
Oleic acid (C18:1)	10.24
Linoleic acid (C18:2)	5.45
Punicic acid (C18:3)	73.21
SFA	8.29
UFA	90.12

### 3.2. Crude protein of sunflower meal protein concentrate

The protein content of SMPC was found to be 55.19 ± 1.27%. In a similar study, Gandhi, Jha (30) observed sunflower meal to have 57.4% protein.

### 3.3. Amino acid characterization

The amino acid profile of SMPC was shown in Supplementary Table S4, in which arginine was 2.59%, histidine 0.97%, isoleucine 1.35%, leucine 1.96%, lysine 0.99%, methionine 0.44%, cysteine 1.22%, phenylalanine 1.55%, threonine 1.85%, valine 1.19%, aspartic acid + asparagine 1.09%, serine 0.49%, glutamic acid + glutamine 1.37%, glycine 0.45%, alanine 0.48%, tyrosine 0.56% and proline was 1.05%. The amino acid profiles may vary based on cultivar varieties, industrial processes and crop/pest control methods (31). While studying the bioactivity potential of industrial sunflower protein ethanol-wash solute, Ivanova, Ivanov (32) observed that leucine and isoleucine had the highest value of all essential amino acids, at 1.16 and 1.40%, respectively.

### 3.4. Proximate analyses of high nutria omega 5 cookies

The higher protein content of 13.36 ± 0.22% was seen in T_5_ (25% SMPC and 25% PSO), while the lower value of 7.09 ± 0.12% was found in T_0_ at 0 days, which diminished as the extent of SMPC and PSO diminished with storage days (Table 2). The results proved that SMPC and PSO show significantly more protein percentages than the control. In a similar study, the protein content of sunflower meal protein concentrates was 57.4%, as described by Gandhi, Jha (30) and 19.60% protein in PSO. In newly prepared cookies, the protein percentage significantly reduced between 6.98 and 10.93% following 60 days (*p* ≤ 0.05). This reduction in the protein substance of cookies amid storage is because of the retention of dampness from the environment. The different proteins found in flour can alter during food processing and storage due to protein cross-linking, protein-carbohydrate interactions, and protein denaturation. Non-enzymatic reactions may also result in food deterioration and shorten the shelf life of foods (33). The results from Table 3 SI indicate that SMPC and PSO fortification may help improve protein content status.

**Table 2:** Means for the effect of treatment and storage intervals on the proximate parameters of high nutria cookies.

Proximate parameters	Treatment	Days				
		0	15	30	45	60
Protein content (%)	T_0_	7.09 ± 0.12	7.07 ± 0.08	7.03 ± 0.08	7.01 ± 0.03	6.98 ± 0.03
	T_1_	7.39 ± 0.11	7.32 ± 0.12	7.18 ± 0.12	7.14 ± 0.10	7.11 ± 0.08
	T_2_	9.36 ± 0.12	9.28 ± 0.13	9.21 ± 0.10	9.19 ± 0.12	9.13 ± 0.15
	T_3_	10.64 ± 0.24	10.49 ± 0.22	10.48 ± 0.14	10.36 ± 0.08	10.35 ± 0.13
	T_4_	11.58 ± 0.24	11.11 ± 0.18	10.84 ± 0.22	10.76 ± 0.12	10.48 ± 0.19
	T_5_	13.36 ± 0.22	12.97 ± 0.22	12.39 ± 0.25	11.53 ± 0.22	10.93 ± 0.24
Fat content (%)	T_o_	15.32 ± 0.26	15.17 ± 0.27	15.12 ± 0.34	15.06 ± 0.35	14.95 ± 0.26
	T_1_	15.51 ± 0.33	15.36 ± 0.23	15.23 ± 0.22	15.30 ± 0.25	15.24 ± 0.42
	T_2_	16.76 ± 0.32	16.66 ± 0.23	16.56 ± 0.28	16.49 ± 0.36	16.42 ± 0.24
	T_3_	17.67 ± 0.38	17.54 ± 0.24	17.56 ± 0.25	17.52 ± 0.27	17.41 ± 0.36
	T_4_	18.72 ± 0.34	18.41 ± 0.39	18.11 ± 0.37	18.14 ± 0.26	17.89 ± 0.33
	T_5_	19.42 ± 0.35	19.33 ± 0.32	19.13 ± 0.31	18.99 ± 0.47	18.91 ± 0.27
Textural hardness (N)	T_0_	18.05 ± 0.71	17.87 ± 0.71	17.55 ± 0.30	16.92 ± 0.59	14.85 ± 0.56
	T_1_	14.81 ± 0.49	14.53 ± 0.28	14.43 ± 0.27	14.15 ± 0.62	13.87 ± 0.51
	T_2_	17.55 ± 0.38	17.17 ± 0.47	16.95 ± 0.36	16.65 ± 0.45	15.76 ± 0.44
	T_3_	20.18 ± 0.26	19.65 ± 0.46	19.26 ± 0.32	19.10 ± 0.74	18.72 ± 0.45
	T_4_	21.82 ± 0.29	21.68 ± 0.59	21.21 ± 0.51	20.96 ± 0.64	20.73 ± 0.62
	T_5_	24.98 ± 0.37	24.62 ± 0.56	24.53 ± 0.37	24.34 ± 0.72	23.82 ± 0.62
Spread factor	T_o_	52.32 ± 0.66	52.52 ± 0.54	52.62 ± 0.59	52.64 ± 0.59	52.59 ± 0.55
	T_1_	53.33 ± 0.68	53.73 ± 0.61	53.82 ± 0.71	53.87 ± 0.59	53.92 ± 0.59
	T_2_	53.15 ± 0.67	54.22 ± 0.59	55.08 ± 0.61	54.81 ± 0.74	55.01 ± 0.52
	T_3_	54.27 ± 0.71	54.51 ± 0.66	54.44 ± 0.67	55.28 ± 0.66	55.38 ± 0.55
	T_4_	54.54 ± 0.51	54.28 ± 0.63	54.34 ± 0.67	54.92 ± 0.59	54.80 ± 0.63
	T_5_	54.94 ± 0.62	54.17 ± 0.64	53.75 ± 0.52	54.42 ± 0.64	54.17 ± 0.53
Energy value (kcal/100 g)	T_o_	439.5 ± 9.6	438.8 ± 10.1	431.4 ± 11.1	425.7 ± 10.2	423.5 ± 11.5
	T_1_	394.2 ± 8.8	392.6 ± 11.4	390.5 ± 11.7	385.4 ± 9.3	386.3 ± 10.5
	T_2_	417.8 ± 11.9	414.6 ± 10.3	409.3 ± 10.2	408.1 ± 11.7	402.2 ± 9.3
	T_3_	443.6 ± 10.5	440.2 ± 10.7	439.2 ± 11.7	435.5 ± 11.1	432.2 ± 11.8
	T_4_	474.4 ± 10.5	474.9 ± 11.7	470.8 ± 8.9	468.2 ± 10.2	467.3 ± 11.9
	T_5_	494.9 ± 9.5	495.0 ± 8.8	493.9 ± 9.9	491.1 ± 10.5	487.4 ± 8.9

**Table 3:** Mean values for protein quantity evaluation in rats.

Groups	Total food intake (g)	Weight gain (g)	Protein in feed (%)	Protein consumed (g)	Protein Efficiency Ratio	Net Protein Ratio
G_0_	616.23 ± 25.72	101.96 ± 09.72	19.37 ± 0.54	119.31 ± 5.66	0.85 ± 0.08	0.76 ± 0.10
G_1_	473.76 ± 16.32	−10.96 ± 03.43	0.17 ± 0.04	0.80 ± 0.05	NA	NA
G_2_	529.55 ± 28.11	160.02 ± 12.72	21.42 ± 0.62	113.31 ± 8.36	1.41 ± 0.09	1.31 ± 0.13
G_3_	548.32 ± 32.88	188.68 ± 16.92	24.36 ± 1.02	133.49 ± 7.53	1.66 ± 0.11	1.33 ± 0.09
G_4_	501.71 ± 21.67	97.32 ± 08.87	19.57 ± 0.38	98.04 ± 4.55	0.99 ± 0.06	0.88 ± 0.07

### 3.5. Crude fat of high nutria omega 5 cookies

The mean values of fat content (%) in High Nutria Omega 5 cookies with different levels of SPMC and PSO are shown in Table 2. A gradual decline was observed in the fat content of the treatments as the storage time increased. This effect was most notably observed in T_5,_ where the fat content reduced from 19.42 ± 0.35% at 0 days to 18.91 ± 0.27% at 60 days of storage time. This decline was less aggressive in treatments with lower PSO and SMPC levels. Since PSO has a high amount of polyunsaturated fatty acids and shortening mostly has a higher amount of saturated fats, this may explain why the declination was more linear in treatments with a higher amount of PSO owing to the lower stability of unsaturated fats. A significant variation level was observed among treatment and storage groups (*p* ≤ 0.05). According to Sharif, Butt (34), this reduction in crude fat during the storage of cookies may be due to the absorption of humidity in cookies from the climate and the oxidation of unsaturated fats breaking down the free unsaturated fat arrangement.

### 3.6. Textural analysis

Textural profile is an imperative parameter for assessing physical nature of food especially baked goods. The means of high nutria cookies for the texture of different treatments appeared in Table 2. The textural hardness was observed to significantly decrease as the storage days increased (*p* ≤ 0.05), which may be attributed to the incorporation of moisture from the atmosphere leading to an increase in moisture content and eventually adding to the dampness of the cookies, which may have gradually reduced the hardness of the cookie treatments. A similar study by Giuffrè et al. (35) also revealed that Cantuccini Biscuits prepared by replacement of shortening with extra virgin olive oil had a decreasing trend in both hardness and fracturability value over 12 months.

### 3.7. Spread factor

The spread factor varied from 52.32 ± 0.66 to 55.38 ± 0.55 in cookies of all treatments. Results reveal that the spared factor of high nutria cookies showed significant variation (*p* ≤ 0.05) with a rise in SMPC and PSO levels for each treatment. The means for the spread factor of baked cookies are mentioned in Table 2. Ahmad et al. (36) observed a similar trend of values while studying the effect of additives on gluten development in cookie dough. The spread factor of cookies from different mills was 40.35 ± 0.620 and 57.66 ± 0.543. Chappalwar et al. (37) reported the same result but observed no consistent trend in the spread factor of the products. The result of this research is in relation to studies explicated by Claughton and Pearce (38), where they detected a slightly non-significant increase in the spread factor of HNO5 equipped from flour comprising 20% sunflower isolate.

### 3.8. Energy value

The energy value decreased significantly (*p* ≤ 0.05) as the storage time elapsed to 60 days. Overall, the lowest energy value was observed in T_1_ (386.3 ± 10.5 kcal/100 g), and the highest energy value was observed in T_5_ (487.4 ± 8.9 kcal/100 g) after 60 days as shown in Table 2. This apparent decline in energy value may be attributed to the oxidation of oil in cookies and moisture gain from the surrounding resulting in lower protein content.

### 3.9. Characterization of conjugated linolenic acid (Omega-5 punicic acid)

In the freshly prepared cookies, the content of punicic acid ranged from 3.77 to 16.14% on the 0th day. Over a period of 60 days, this percentage gradually decreased to a range of 3.33 and 14.37%. As apparent from the mean values in Supplementary Table S5, a significant decline in the punicic acid of high nutria cookies can be seen after a storage time of 60 days. A considerable variation was observed in both treatment levels and the storage interval of the High Nutria Omega 5 cookies (*p* ≤ 0.05). According to Sharif et al. (39), a reduction in fatty acid contents during the storage of cookies could result from moisture absorption in cookies from the environment and oxidation of unsaturated fats bringing about free fatty acids. Punicic acid is generally known as CLnA (conjugated linolenic acid) and PSO generally contains 78% punicic acid (40).

### 3.10. Selection of best treatment

The consequent data obtained from the proximate, physicochemical, storage and sensory evaluation revealed that T_3_ (15% PSO + 15% SMPC) was most suitable for further evaluation (Supplementary Figures S1–S5).

### 3.11. Serum analyses

#### 3.11.1. Liver functioning test

In our investigation, malnourished groups of rats had significantly higher blood levels of the liver function indicators ALT, AST, and ALP than normal control rats. The rationale for the significant increase in serum levels of aminotransferases in the current study’s starved rats may be that these enzymes escaped into circulation following hepatic damage in the context of prolonged fasting (41). According to the results, administering several experimental diets considerably improved the blood levels of the enzymes ALT, AST, and ALP (Table 4). These reported benefits might result from SMPC and PSO’s flavonoids, terpenoids, and alkaloids, which have antioxidant and hepatoprotective activities. Along with their ability to stabilize membranes, these phyto-constituents are known to inhibit the release of intracellular liver enzymes. These outcomes are backed up by one of the earlier investigations, which used high carbohydrate, high protein, and high-fat diets, which were re-fed to rats after 3 days of starvation. After following a high fat and high protein diet, the levels of ALT and AST returned to the normal range as per their respective reference values. However, in the case of a high carbohydrate diet, liver functioning tests showed significantly higher values compared to the reference values (42).

**Table 4:** Means for the effect of groups and days on the liver functioning tests of rats.

	Group	Days			
		0	15	30	45
ALT (U/L)	G_0_	39.41 ± 1.78	40.01 ± 1.22	39.12 ± 1.79	38.37 ± 1.89
	G_1_	44.45 ± 1.83	43.39 ± 1.04	42.62 ± 1.52	42.76 ± 1.98
	G_2_	45.37 ± 1.78	42.02 ± 1.78	38.62 ± 1.93	36.32 ± 1.15
	G_3_	44.20 ± 1.66	41.62 ± 1.01	37.18 ± 1.06	34.46 ± 1.63
	G_4_	45.14 ± 1.59	43.12 ± 1.77	40.09 ± 1.12	38.31 ± 1.97
AST (U/L)	G_0_	135.21 ± 02.98	134.35 ± 02.43	134.05 ± 02.98	133.97 ± 02.73
	G_1_	142.50 ± 02.84	142.35 ± 02.49	141.19 ± 03.04	141.07 ± 03.04
	G_2_	141.03 ± 01.27	137.41 ± 02.44	134.37 ± 02.31	131.20 ± 02.37
	G_3_	142.25 ± 01.59	140.41 ± 02.79	137.04 ± 02.15	135.43 ± 02.52
	G_4_	143.09 ± 03.56	142.95 ± 03.14	138.63 ± 02.42	132.19 ± 02.60
ALP (U/L)	G_0_	54.63 ± 1.89	51.47 ± 1.39	53.45 ± 1.44	52.42 ± 1.98
	G_1_	64.54 ± 1.91	63.70 ± 1.97	61.78 ± 1.45	59.47 ± 1.09
	G_2_	65.61 ± 1.14	62.61 ± 1.42	60.43 ± 1.11	57.60 ± 1.77
	G_3_	64.59 ± 1.15	61.27 ± 1.38	59.62 ± 1.06	54.51 ± 1.46
	G_4_	63.68 ± 1.65	62.65 ± 1.79	58.68 ± 1.86	55.68 ± 1.31

#### 3.11.2. Renal functioning tests

Results have shown that mean urea and creatinine in malnourished rats were significantly increased after starvation (G_1_). Treatment of malnourished rats with HNO5 cookies, SMPC and PSO significantly decreased the level of urea and creatinine from day 0 to the 45th day of the experiment (Table 5). Moreover, experimental diets in treated groups G_2_, G_3_, and G_4_ from day 0 to day 45 also restored the renal functioning in the reference range affected due to malnutrition compared to the control group (G_0_). This finding is consistent with previous research involving starved rats to determine suitable diet recovery with macronutrients.

**Table 5:** Means for the effect of groups and days on the renal functioning tests of rats.

	Group	Days			
		0	15	30	45
Urea level (mg/dL)	G_0_	22.12 ± 0.60	21.93 ± 0.56	21.41 ± 0.49	21.08 ± 0.54
	G_1_	25.97 ± 0.54	26.01 ± 0.26	24.64 ± 0.91	24.30 ± 0.49
	G_2_	26.40 ± 0.49	25.03 ± 0.97	22.31 ± 0.39	20.65 ± 0.41
	G_3_	26.08 ± 0.91	25.12 ± 0.27	23.16 ± 0.44	21.06 ± 0.63
	G_4_	25.94 ± 0.56	24.78 ± 0.47	22.78 ± 0.32	20.43 ± 0.44
Creatinine level (mg/dL)	G_0_	0.55 ± 0.011	0.52 ± 0.012	0.51 ± 0.010	0.49 ± 0.013
	G_1_	0.84 ± 0.016	0.82 ± 0.012	0.81 ± 0.016	0.80 ± 0.013
	G_2_	0.80 ± 0.012	0.75 ± 0.014	0.68 ± 0.019	0.58 ± 0.010
	G_3_	0.85 ± 0.015	0.73 ± 0.010	0.68 ± 0.004	0.56 ± 0.002
	G_4_	0.66 ± 0.013	0.58 ± 0.008	0.51 ± 0.009	0.54 ± 0.004

Creatinine and urea are metabolism’s nitrogenic end products. Urea is the primary metabolite of the recycling of tissue and dietary protein (43). Factors like dehydration and low-protein diets are responsible for an increased level of urea, whereas creatinine is more particular to the kidney (44). Hence, research findings show that administration of PSO, SMPC and particularly combined in HNO5 cookies inhibited serum urea and creatinine while enhancing renal function.

#### 3.11.3. Level of punicic acid

**Figure 1:**
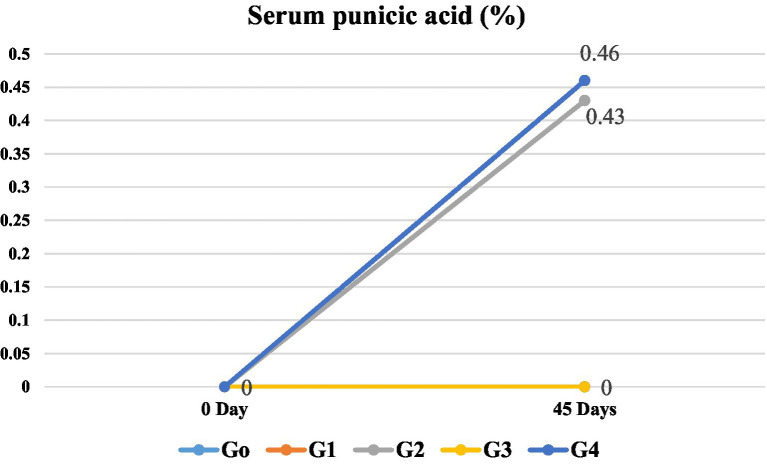
Effect of groups and days on the serum punicic acid (% fatty acid) in rats.

Results indicated a significant increase in serum punicic acid with respect to days and treatments. Increased levels of punicic acid were observed in groups G_2_ and G_4_ at the end of the study, with levels of 0.43 and 0.46% of fatty acids, respectively. At the same time, G_0,_ G_1_ and G_3_ had no value of punicic acid observed as there was no source of punicic acid in their diets designed (Figure 1). Punicic acid significantly (*p* ≤ 0.05) varied among different groups. These results are also supported by a study by Yuan et al. (2009) on punicic acid metabolism and its retaining in blood in young, healthy males. In another study, 30 males were randomly divided into two groups after 7 days of adaptation period with sunflower seed kernels. The control group was fed sunflower seed kernels, while the second group was with punicic acid rich *Trichosanthes kirilowii* (TK) seeds with 3 g of punicic acid, and the study period was for 28 days. Serum punicic acid was evaluated at the start and end of the trial in both groups. Punicic acid was zero on the first day of study in all the experimental groups; however, it raised significantly in the HNO5 cookies group, and PSO treated group with punicic acid-rich seeds but remained 0 in the normal and positive control group. It was incorporated into human tissues, and some of it was metabolized into cis9, trans11-18:2.

#### 3.11.4. Serum proteins

Long-term starvation significantly reduced the metabolic stress indicators such as total serum proteins, albumin, globulin, and A/G ratio in malnourished rats compared to positive control and negative control (normal group; Table 6). The ratio of protein production to catabolism inside the body determines the number of tissue proteins. Increased protein catabolism may cause a drop in total proteins and albumin levels (45). According to the findings of our study, experimental diets restored serum total proteins, albumin, globulin, and the A/G ratio compared to the positive control group. Compared to the positive control, the experimental diets HNO5 cookies and SMPC restored the blood total protein level more successfully. Protein content in SMPC and HNO5 cookies has been determined to be 53.19 and 10.35%, respectively, and might aid in restoring the blood total protein level. Malnourished rats given with SMPC and HNO5 cookies had higher blood total proteins, albumin, globulin, and A/G ratios, which may have been caused by insulin-stimulated amino acid uptake, enhanced protein synthesis, and decreased protein breakdown, and a similar trend has been observed by Vidhya and Udayakumar (46).

**Table 6:** Means for the effect of groups and days on the serum proteins of rats.

	Group	Days			
		0	15	30	45
Total serum proteins (g/dL)	G_0_	5.92 ± 0.23	5.93 ± 0.15	5.94 ± 0.13	5.96 ± 0.14
	G_1_	5.06 ± 0.12	4.97 ± 0.17	4.90 ± 0.19	4.85 ± 0.17
	G_2_	5.04 ± 0.14	5.97 ± 0.26	6.44 ± 0.13	7.03 ± 0.18
	G_3_	5.07 ± 0.15	5.93 ± 0.13	6.81 ± 0.14	7.62 ± 0.25
	G_4_	5.06 ± 0.16	5.04 ± 0.15	5.02 ± 0.16	5.01 ± 0.14
Serum albumin (g/dL)	G_0_	3.71 ± 0.06	3.72 ± 0.04	3.74 ± 0.03	3.76 ± 0.01
	G_1_	2.21 ± 0.02	2.15 ± 0.05	2.09 ± 0.01	2.05 ± 0.04
	G_2_	2.20 ± 0.04	2.85 ± 0.07	3.18 ± 0.02	3.82 ± 0.08
	G_3_	2.24 ± 0.07	2.87 ± 0.06	3.34 ± 0.09	3.92 ± 0.07
	G_4_	2.22 ± 0.03	2.21 ± 0.05	2.19 ± 0.04	2.18 ± 0.02
Serum globulins (g/dL)	G_0_	2.21 ± 0.18	2.21 ± 0.13	2.20 ± 0.15	2.20 ± 0.15
	G_1_	2.85 ± 0.13	2.82 ± 0.15	2.81 ± 0.13	2.80 ± 0.19
	G_2_	2.84 ± 0.19	3.12 ± 0.14	3.26 ± 0.16	3.21 ± 0.13
	G_3_	2.83 ± 0.14	3.06 ± 0.19	3.47 ± 0.11	3.70 ± 0.11
	G_4_	2.84 ± 0.12	2.83 ± 0.11	2.83 ± 0.14	2.83 ± 0.15
A/G ratio	G_0_	1.67 ± 0.01	1.68 ± 0.01	1.70 ± 0.02	1.71 ± 0.09
	G_1_	0.77 ± 0.02	0.76 ± 0.02	0.74 ± 0.01	0.73 ± 0.02
	G_2_	0.77 ± 0.02	0.91 ± 0.09	0.97 ± 0.09	1.19 ± 0.02
	G_3_	0.79 ± 0.09	0.93 ± 0.01	0.96 ± 0.01	1.05 ± 0.01
	G_4_	0.78 ± 0.01	0.78 ± 0.02	0.77 ± 0.02	0.77 ± 0.01

#### 3.11.5. Protein quality evaluation

The protein efficiency ratio is the gain in body weight per unit protein intake, and the net protein ratio is defined as the ratio of the sum of weight gain and average weight loss to that of protein intake of the test protein group after 28 days. Table 3 shows mean squares for the (NRP) protein quality and protein efficiency ratio (PER) of the test diets. All protein quality parameters (PER and NPR) varied significantly across the experimental diets. Apart from the control, SMPC-based diets (G_2_ and G_3_) had the highest values compared to the PSO containing diet after 45 days of study duration. Improved protein quality was observed in experimental diets containing SMPC. Rats on a diet devoid of protein underperformed regarding growth, food intake, and weight loss. The same conclusions were reported by Ekpo (47) as both protein consumption and food intake significantly influence growth while low protein leads to decreased food intake and reduced development. However, according to other research, animals given ordinary maize exhibited consistent development (48). Halimatul et al. (49) also observed a similar increasing trend in PER and NPR of Sprague Dawley rats that were fed with Roselle (*Hibiscus sabdariffa* L.) seeds.

#### 3.11.6. Lipid profile

Overall, lipid profile measurements showed a statistically significant difference between all treatment groups after study completion. Diets containing PSO and HNO5 cookies reduced cholesterol significantly compared to control cookies. Concerning serum lipids profile, malnourished rats presented different TC results depending on the experimental diet containing high protein (G_3_) than the positive control (G_1_) protein-free group. Although, chronic malnutrition causes endocrine changes in metabolic profile disorders. The decrease in triglyceride level in the HNO5 cookies group compared to control cookies is shown in Table 7. Triglyceride levels of rat’s lipid profile in HNO5 and PSO treated groups were significantly improved, indicating that the oil is a potent cardio-protective treatment. This effect was attributed to the super conjugated linolenic acid, or punicic acid, that is contained in pomegranate seed oil. Widyastuti et al. (50) revealed that rats with PEM had higher levels of total cholesterol, triglycerides, and LDL, whereas their HDL levels dropped when compared to the healthy control group. HDL cholesterol levels were significantly increased in rats fed on SMPC and PSO diet with a significant decrease in LDL cholesterol levels. Pasqua et al. (51) also delineated results for evaluating the safety and beneficial outcomes of a conventional diet supplemented with whey-derived protein puddings and hemp seed oil to counteract malnutrition; no significant variations were discovered in plasma total, LDL and HDL cholesterol as well as in glycaemia.

**Table 7:** Means for the effect of groups and days on the lipid profile of rats.

	Group	Days			
		0	15	30	45
Total Cholesterol (mg/dL)	G_0_	59.71 ± 1.99	60.67 ± 1.96	59.54 ± 2.01	58.57 ± 1.97
	G_1_	67.34 ± 1.39	70.36 ± 1.87	76.38 ± 2.08	87.45 ± 2.07
	G_2_	69.02 ± 1.41	66.68 ± 1.07	64.70 ± 1.14	62.23 ± 1.87
	G_3_	69.56 ± 1.20	65.38 ± 1.86	63.27 ± 1.09	60.27 ± 1.02
	G_4_	68.27 ± 1.71	66.15 ± 2.00	62.21 ± 1.19	59.04 ± 1.07
Triglyceride (mg/dL)	G_0_	73.45 ± 1.74	72.09 ± 1.31	72.14 ± 1.09	71.50 ± 1.96
	G_1_	67.40 ± 1.46	69.34 ± 1.37	73.19 ± 1.57	77.25 ± 1.12
	G_2_	76.24 ± 1.35	74.27 ± 1.08	72.25 ± 1.75	69.36 ± 1.77
	G_3_	78.65 ± 1.60	76.32 ± 1.06	74.56 ± 2.09	72.63 ± 1.43
	G_4_	76.52 ± 1.26	73.06 ± 1.16	70.28 ± 1.51	66.58 ± 1.42
LDL (mg/dL)	G_0_	50.30 ± 1.07	48.42 ± 1.47	49.29 ± 1.61	47.40 ± 1.50
	G_1_	59.21 ± 1.60	62.36 ± 1.62	65.21 ± 1.16	68.23 ± 1.63
	G_2_	58.15 ± 1.66	56.28 ± 1.37	55.17 ± 1.34	54.27 ± 1.52
	G_3_	59.04 ± 1.25	57.34 ± 1.78	54.09 ± 1.63	51.36 ± 1.90
	G_4_	58.36 ± 1.75	56.20 ± 1.42	53.45 ± 1.04	49.48 ± 1.68
HDL (mg/dL)	G_0_	44.75 ± 1.39	45.15 ± 1.68	46.16 ± 1.29	45.13 ± 1.22
	G_1_	40.67 ± 1.53	38.23 ± 1.37	37.21 ± 1.36	35.03 ± 1.65
	G_2_	40.45 ± 1.57	41.09 ± 1.93	42.32 ± 1.47	45.10 ± 1.62
	G_3_	40.53 ± 1.54	42.15 ± 1.55	43.10 ± 1.51	46.21 ± 1.01
	G_4_	39.23 ± 1.80	44.20 ± 1.52	47.21 ± 1.74	50.53 ± 1.43

#### 3.11.7. Serum antioxidants

Statistical analysis revealed significant (*p* ≤ 0.05) variation in groups and with respect to study duration on serum TAC content. Endogenous antioxidant enzymes significantly affect the body’s defense against free radicals, and increased total antioxidant status after being treated with experimental diets ensures a safe and effective strategy in alleviating oxidative stress, most probably due to PSO antioxidant potential. Ali et al. (52) also determined that in both usually fed (NF) and protein-malnourished rats, treatment of AlCl3 dramatically raised MDA and lowered SOD and TAC activity. However, compared to NF rats, protein-malnourished rats had a significantly higher level of MDA and a lower level of SOD and TAC activity (Table 8).

**Table 8:** Means for the effect of groups and days on the TAC and TOS of rats.

	Group	Days			
		0	15	30	45
TAC (mmol/L)	G_0_	2.18 ± 0.02	2.19 ± 0.04	2.17 ± 0.05	2.20 ± 0.07
	G_1_	2.03 ± 0.08	2.09 ± 0.05	2.13 ± 0.07	2.14 ± 0.04
	G_2_	2.01 ± 0.01	2.76 ± 0.07	2.89 ± 0.01	2.96 ± 0.02
	G_3_	2.01 ± 0.09	2.75 ± 0.01	2.83 ± 0.04	2.88 ± 0.05
	G_4_	2.02 ± 0.01	2.56 ± 0.09	2.72 ± 0.02	2.87 ± 0.03
TOS (μmol/L)	G_0_	7.04 ± 0.12	7.04 ± 0.13	7.07 ± 0.16	7.06 ± 0.15
	G_1_	8.40 ± 0.15	8.46 ± 0.19	8.44 ± 0.14	8.43 ± 0.13
	G_2_	8.70 ± 0.18	8.56 ± 0.12	7.84 ± 0.17	6.62 ± 0.16
	G_3_	8.80 ± 0.15	7.26 ± 0.11	7.16 ± 0.18	6.97 ± 0.14
	G_4_	8.36 ± 0.17	7.21 ± 0.13	7.18 ± 0.15	6.67 ± 0.12

## 4. Conclusion

the incorporation of pomegranate seed oil (PSO) and sunflower meal protein concentrate (SMPC) in the production of High Nutria Omega 5 (HNO5) cookies has yielded promising results. The study demonstrated that cookies containing 15% PSO and 15% SMPC displayed stability in various physicochemical and sensory evaluations. Furthermore, the inclusion of punicic acid from PSO and SMPC in the HNO5 cookies showed significant improvements in overall health and reduced the negative effects of starvation in rats, leading to enhanced body weight and overall well-being. The positive outcomes of this study indicate that these byproducts can be valuable resources in addressing malnutrition, potentially replacing commonly used ingredients like vegetable shortening and flour. By utilizing these waste byproducts in the food industry, a sustainable approach can be adopted, minimizing waste generation and generating new functional food products. The findings highlight the potential of PSO and SMPC as effective ingredients for the development of healthier and more sustainable food options. Therefore, it is crucial to further explore and develop these byproducts for the betterment of our health and the environment. Continued research and development in this area will contribute to a healthier and more sustainable future.

## Data availability statement

The original contributions presented in the study are included in the article/Supplementary material, further inquiries can be directed to the corresponding authors.

## Ethics statement

The animal study was reviewed and approved by Institutional biosafety committees (IBC) University of Agriculture, Faisalabad.

## Author contributions

NI and MAS conceived the work, collected raw materials, carried out experimentations, analyzed and interpreted data, and wrote the article. MRK assisted in experimentations and read the article. MAS, MNF, and MRK supervised the work and review the article. All authors have approved the final article.

## Conflict of interest

The authors declare that the research was conducted in the absence of any commercial or financial relationships that could be construed as a potential conflict of interest.

## Publisher’s note

All claims expressed in this article are solely those of the authors and do not necessarily represent those of their affiliated organizations, or those of the publisher, the editors and the reviewers. Any product that may be evaluated in this article, or claim that may be made by its manufacturer, is not guaranteed or endorsed by the publisher.
