# Supplementary material for: Development and bioassessment of high nutria-omega 5 cookies through animal modeling
Source: Front Nutr. 2023 Jun 30;10:1199645. doi: 10.3389/fnut.2023.1199645 (PMC10348480; doi:10.3389/fnut.2023.1199645)
Supplement: Supplementary file 1 [file Data_Sheet_1.docx]

**S Table 1: Treatment plan for High Nutria-Omega 5 cookies**

| **Treatments** | **Pomegranate seed oil (%)** | **Flour (%)** | **Sunflower meal protein concentrate (%)** | **Shortening (%)** |
| --- | --- | --- | --- | --- |
| **T_0_ (Control)** | - | 100 | - | 100 |
| **T_1_ (05% PSO + 05% SMPC)** | 5 | 95 | 5 | 95 |
| **T_2_ (10% PSO + 10% SMPC)** | 10 | 90 | 10 | 90 |
| **T_3_ (15% PSO + 15% SMPC)** | 15 | 85 | 15 | 85 |
| **T_4_ (20% PSO + 20% SMPC)** | 20 | 80 | 20 | 80 |
| **T_5_ (25% PSO + 25% SMPC)** | 25 | 75 | 25 | 75 |

**S Table 2.** Treatment plan for the efficacy study

| **Groups** | **Treatment** |
| --- | --- |
| **G_0_** | Normal feed |
| **G_1_** | Starvation+ Normal feed + Control cookies |
| **G_2_** | Starvation+ Normal feed + High Nutria Omega 5 Cookies |
| **G_3_** | Starvation+ Normal feed + SMPC |
| **G_4_** | Starvation+ Normal feed + PSO |

**S Table 3.** Experimental Diet Plan for Male Sprague Dawley (SD) strain rats

| **Ingredients** | **Normal feed (%)** | **Normal feed + Control cookies (%)** | **Normal feed + HNO5 cookies (%)** | **Normal feed + SMPC** | **Normal feed + PSO** |
| --- | --- | --- | --- | --- | --- |
| **Control cookies** | - | 15 |  |  | - |
| **HNO5 Cookies** | - | - | 15 |  |  |
| **SMPC** | - | - |  | 15 |  |
| **PSO** | - | - |  |  | 15 |
| **Corn starch** | 65 | 70 | 50 | 50 | 50 |
| **Casein** | 20 | 0 | 20 | 20 | 20 |
| **Cellulose** | 10 | 10 | 10 | 10 | 10 |
| **Salt mixture** | 4 | 4 | 4 | 4 | 4 |
| **Vitamins** | 1 | 1 | 1 | 1 | 1 |
| **Total** | 100 | 100 | 100 | 100 | 100 |

**S Table 4.** Resultant values for Amino acid (%) of SMPC.

| **Sr. No.** | **Name of amino acid** | **% of DM** |
| --- | --- | --- |
| 1 | Cysteine | 1.22 |
| 2 | Methionine | 0.44 |
| 3 | Aspartic Acid + Asparagine | 1.09 |
| 4 | Threonine | 1.85 |
| 5 | Serine | 0.49 |
| 6 | Glutamic Acid + Glutamine | 1.37 |
| 7 | Glycine | 0.45 |
| 8 | Alanine | 0.48 |
| 9 | Valine | 1.19 |
| 10 | Isoleucine | 1.35 |
| 11 | Leucine | 1.96 |
| 12 | Phenylalanine | 1.55 |
| 13 | Histidine | 0.97 |
| 14 | Lysine | 0.99 |
| 15 | Tyrosine | 0.56 |
| 16 | Arginine | 2.59 |
| 17 | Proline | 1.05 |

**S Table 5.** Effect of treatment and storage intervals on the level of Punicic acid (%) of high nutria cookies

| **Treatment** | **0 Day** | **60 Days** |
| --- | --- | --- |
| **T_0_** | 0.00 | 0.00 |
| **T_1_** | 3.77 | 3.33 |
| **T_2_** | 7.01 | 6.54 |
| **T_3_** | 10.61 | 10.09 |
| **T_4_** | 13.86 | 12.83 |
| **T_5_** | 16.14 | 14.37 |

Means that do not share same letter are significantly different (P > 0.05). Data expressed as mean ± S.E. (n= 3).

**S Figure 1: Means for the effect of treatment and storage intervals on the color of high nutria cookies**

**S Figure 2: Means for the effect of treatment and storage intervals on the flavor of high nutria cookies**

**S Figure 3: Means for the effect of treatment and storage intervals on the taste of high nutria cookies**

**S Figure 4: Means for the effect of treatment and storage intervals on the texture of high nutria cookies**

**S Figure 5: Means for the effect of treatment and storage intervals on the overall acceptability of high nutria cookies**
