# Supplementary material for: Development and bioassessment of high nutria-omega 5 cookies through animal modeling
Source: Front Nutr. 2023 Jun 30;10:1199645. doi: 10.3389/fnut.2023.1199645 (PMC10348480; doi:10.3389/fnut.2023.1199645)
Supplement: Supplementary file 2 [file Data_Sheet_2.docx]

**Mean square values for protein content of high nutria cookies**

| **Source** | **DF** | **SS** | **MS** | **F-Value** |
| --- | --- | --- | --- | --- |
| **Treatment** | 5 | 327.738 | 65.5477 | 2544.55** |
| **Storage** | 4 | 6.199 | 1.5496 | 60.16** |
| **Treatment*Storage** | 20 | 8.428 | 0.4214 | 16.36** |
| **Error** | 60 | 1.546 | 0.0258 |  |
| **Total** | 89 | 343.911 |  |  |
| **= Highly significant (P≤0.01) *= Significant (P≤0.05) NS= Non-significant (P≥0.05) | | | | |

**Mean square values for crude fat of high nutria cookies**

| **Source** | **DF** | **SS** | **MS** | **F-Value** |
| --- | --- | --- | --- | --- |
| **Treatment** | 5 | 195.081 | 39.0163 | 392.90** |
| **Storage** | 4 | 1.953 | 0.4882 | 4.92** |
| **Treatment*Storage** | 20 | 0.541 | 0.0270 | 0.27^NS^ |
| **Error** | 60 | 5.958 | 0.0993 |  |
| **Total** | 89 | 203.533 |  |  |
| **= Highly significant (P≤0.01) *= Significant (P≤0.05) NS= Non-significant (P≥0.05) | | | | |

**Mean square values for textural hardness of high nutria cookies**

| **Source** | **DF** | **SS** | **MS** | **F-Value** |
| --- | --- | --- | --- | --- |
| **Treatment** | 5 | 977.85 | 195.571 | 752.68** |
| **Storage** | 4 | 27.12 | 60780 | 26.09** |
| **Treatment*Storage** | 20 | 8.74 | 0.437 | 1.68^NS^ |
| **Error** | 60 | 15.59 | 0.260 |  |
| **Total** | 89 | 1029.39 |  |  |
| **= Highly significant (P≤0.01) *= Significant (P≤0.05) NS= Non-significant (P≥0.05) | | | | |

**Mean square values for spread factor of high nutria cookies**

| **Source** | **DF** | **SS** | **MS** | **F-Value** |
| --- | --- | --- | --- | --- |
| **Treatment** | 5 | 51.366 | 10.2731 | 26.85** |
| **Storage** | 4 | 4.619 | 1.1547 | 3.02* |
| **Treatment*Storage** | 20 | 10.555 | 0.5277 | 1.38^NS^ |
| **Error** | 60 | 22.961 | 0.3827 |  |
| **Total** | 89 | 89.500 |  |  |
| **= Highly significant (P≤0.01) *= Significant (P≤0.05) NS= Non-significant (P≥0.05) | | | | |

**Mean square values for energy value of high nutria cookies**

| **Source** | **DF** | **SS** | **MS** | **F-Value** |
| --- | --- | --- | --- | --- |
| **Treatment** | 5 | 107685 | 21536.9 | 204.29** |
| **Storage** | 4 | 1525 | 381.2 | 3.62* |
| **Treatment*Storage** | 20 | 239 | 12.0 | 0.11^NS^ |
| **Error** | 60 | 6325 | 105.4 |  |
| **Total** | 89 | 115774 |  |  |
| **= Highly significant (P≤0.01) *= Significant (P≤0.05) NS= Non-significant (P≥0.05) | | | | |

**Mean square values for punicic acid levels of high nutria cookies**

| **Source** | **DF** | **SS** | **MS** | **F** |
| --- | --- | --- | --- | --- |
| **Treatment** | 5 | 343.517 | 68.7035 | 362.26** |
| **Storage** | 1 | 1.491 | 1.4911 | 7.86* |
| **Error** | 5 | 0.948 | 0.1897 |  |
| **Total** | 11 | 345.957 |  |  |
| **= Highly significant (P≤0.01) *= Significant (P≤0.05) NS= Non-significant (P≥0.05) | | | | |

**Mean square values for color of high nutria cookies**

| **Source** | **DF** | **SS** | **MS** | **F-Value** |
| --- | --- | --- | --- | --- |
| **Treatment** | 5 | 2.206 | 0.4412 | 1.76^NS^ |
| **Storage** | 4 | 2.412 | 0.6031 | 2.41^NS^ |
| **Treatment*Storage** | 20 | 12.887 | 0.6444 | 2.52** |
| **Error** | 60 | 15.000 | 0.2500 |  |
| **Total** | 89 | 32.506 |  |  |
| **= Highly significant (P≤0.01) *= Significant (P≤0.05) NS= Non-significant (P≥0.05) | | | | |

**Mean square values for flavor of high nutria cookies**

| **Source** | **DF** | **SS** | **MS** | **F-Value** |
| --- | --- | --- | --- | --- |
| **Treatment** | 5 | 14.806 | 2.9612 | 11.84** |
| **Storage** | 4 | 26.537 | 6.6344 | 26.54** |
| **Treatment*Storage** | 20 | 8.787 | 0.4394 | 1.76* |
| **Error** | 60 | 15.000 | 0.2500 |  |
| **Total** | 89 | 65.131 |  |  |
| **= Highly significant (P≤0.01) *= Significant (P≤0.05) NS= Non-significant (P≥0.05) | | | | |

**Mean square values for taste of high nutria cookies**

| **Source** | **DF** | **SS** | **MS** | **F-Value** |
| --- | --- | --- | --- | --- |
| **Treatment** | 5 | 14.48 | 2.8962 | 11.88** |
| **Storage** | 4 | 38.78 | 9.6940 | 39.77** |
| **Treatment*Storage** | 20 | 13.67 | 0.6836 | 2.80** |
| **Error** | 60 | 14.63 | 0.2438 |  |
| **Total** | 89 | 81.55 |  |  |
| **= Highly significant (P≤0.01) *= Significant (P≤0.05) NS= Non-significant (P≥0.05) | | | | |

**Mean square values for texture of high nutria cookies**

| **Source** | **DF** | **SS** | **MS** | **F-Value** |
| --- | --- | --- | --- | --- |
| **Treatment** | 5 | 9.781 | 1.9562 | 7.82** |
| **Storage** | 4 | 25.375 | 6.3437 | 25.37** |
| **Treatment*Storage** | 20 | 12.500 | 0.6250 | 2.50** |
| **Error** | 60 | 15.000 | 0.2500 |  |
| **Total** | 89 | 62.656 |  |  |
| **= Highly significant (P≤0.01) *= Significant (P≤0.05) NS= Non-significant (P≥0.05) | | | | |

**Mean square values for overall acceptability of high nutria cookies**

| **Source** | **DF** | **SS** | **MS** | **F-Value** |
| --- | --- | --- | --- | --- |
| **Treatment** | 5 | 19.63 | 3.9262 | 15.70** |
| **Storage** | 4 | 59.12 | 14.7812 | 59.12** |
| **Treatment*Storage** | 20 | 13.40 | 0.6700 | 2.68** |
| **Error** | 60 | 15.00 | 0.2500 |  |
| **Total** | 89 | 107.16 |  |  |
| **= Highly significant (P≤0.01) *= Significant (P≤0.05) NS= Non-significant (P≥0.05) | | | | |

**Mean square values for total alanine aminotransferase (ALT) of rats**

| **Source** | **DF** | **SS** | **MS** | **F-Value** |
| --- | --- | --- | --- | --- |
| **Group** | 4 | 139.2 | 34.795 | 13.43** |
| **Days** | 3 | 288.4 | 96.122 | 37.09** |
| **Group*Days** | 12 | 118.8 | 9.897 | 3.8** |
| **Error** | 40 | 103.7 | 2.592 |  |
| **Total** | 59 | 650.0 |  |  |
| **= Highly significant (P≤0.01) *= Significant (P≤0.05) NS= Non-significant (P≥0.05) | | | | |

**Mean square values for aspartate aminotransferase (AST) of rats**

| **Source** | **DF** | **SS** | **MS** | **F-Value** |
| --- | --- | --- | --- | --- |
| **Group** | 4 | 400.1 | 100.01 | 14.41** |
| **Days** | 3 | 322.0 | 107.34 | 15.46** |
| **Group*Days** | 12 | 167.7 | 13.98 | 2.01* |
| **Error** | 40 | 277.6 | 6.94 |  |
| **Total** | 59 | 1167.5 |  |  |
| **= Highly significant (P≤0.01) *= Significant (P≤0.05) NS= Non-significant (P≥0.05) | | | | |

**Mean square values for alkaline phosphatase (ALP) of rats**

| **Source** | **DF** | **SS** | **MS** | **F-Value** |
| --- | --- | --- | --- | --- |
| **Group** | 4 | 666.24 | 166.560 | 70.01** |
| **Days** | 3 | 353.33 | 117.776 | 49.50** |
| **Group*Days** | 12 | 93.83 | 7.819 | 3.29** |
| **Error** | 40 | 95.17 | 2.379 |  |
| **Total** | 59 | 1208.56 |  |  |
| **= Highly significant (P≤0.01) *= Significant (P≤0.05) NS= Non-significant (P≥0.05) | | | | |

**Mean square values for urea level of rats**

| **Source** | **DF** | **SS** | **MS** | **F-Value** |
| --- | --- | --- | --- | --- |
| **Group** | 4 | 79.07 | 19.767 | 61.24** |
| **Days** | 3 | 131.70 | 43.899 | 136.00** |
| **Group*Days** | 12 | 35.35 | 2.945 | 9.12* |
| **Error** | 40 | 12.91 | 0.322 |  |
| **Total** | 59 | 259.02 |  |  |
| **= Highly significant (P≤0.01) *= Significant (P≤0.05) NS= Non-significant (P≥0.05) | | | | |

**Mean square values for creatinine level of rats**

| **Source** | **DF** | **SS** | **MS** | **F-Value** |
| --- | --- | --- | --- | --- |
| **Group** | 4 | 0.67866 | 0.169665 | 1196.93** |
| **Days** | 3 | 0.17406 | 0.058020 | 409.31** |
| **Group*Days** | 12 | 0.08394 | 0.006995 | 49.35** |
| **Error** | 40 | 0.00567 | 0.000142 |  |
| **Total** | 59 | 0.94233 |  |  |
| **= Highly significant (P≤0.01) *= Significant (P≤0.05) NS= Non-significant (P≥0.05) | | | | |

**Mean square values for total proteins of rats**

| **Source** | **DF** | **SS** | **MS** | **F-Value** |
| --- | --- | --- | --- | --- |
| **Group** | 4 | 20.141 | 5.03515 | 176.21** |
| **Days** | 3 | 6.099 | 2.03297 | 71.15** |
| **Group*Days** | 12 | 11.258 | 0.93819 | 32.83** |
| **Error** | 40 | 1.143 | 0.02857 |  |
| **Total** | 59 | 38.641 |  |  |
| **= Highly significant (P≤0.01) *= Significant (P≤0.05) NS= Non-significant (P≥0.05) | | | | |

**Mean square values for serum albumin of rats**

| **Source** | **DF** | **SS** | **MS** | **F-Value** |
| --- | --- | --- | --- | --- |
| **Group** | 4 | 21.727 | 5.43184 | 2130.13** |
| **Days** | 3 | 3.141 | 1.04705 | 410.61** |
| **Group*Days** | 12 | 5.577 | 0.46477 | 182.26** |
| **Error** | 40 | 0.102 | 0.00255 |  |
| **Total** | 59 | 30.547 |  |  |
| **= Highly significant (P≤0.01) *= Significant (P≤0.05) NS= Non-significant (P≥0.05) | | | | |

**Mean square values for serum globulins of rats**

| **Source** | **DF** | **SS** | **MS** | **F-Value** |
| --- | --- | --- | --- | --- |
| **Group** | 4 | 7.8682 | 1.96705 | 90.77** |
| **Days** | 3 | 0.5084 | 0.16948 | 7.82** |
| **Group*Days** | 12 | 1.2002 | 0.10002 | 4.62** |
| **Error** | 40 | 0.8668 | 0.02167 |  |
| **Total** | 59 | 10.4436 |  |  |
| **= Highly significant (P≤0.01) *= Significant (P≤0.05) NS= Non-significant (P≥0.05) | | | | |

**Mean square values for A/G ratio of rats**

| **Source** | **DF** | **SS** | **MS** | **F-Value** |
| --- | --- | --- | --- | --- |
| **Group** | 4 | 7.11684 | 1.77921 | 977.59** |
| **Days** | 3 | 0.13672 | 0.04557 | 25.04** |
| **Group*Days** | 12 | 0.24900 | 0.02075 | 11.40** |
| **Error** | 40 | 0.07280 | 0.00182 |  |
| **Total** | 59 | 7.57536 |  |  |
| **= Highly significant (P≤0.01) *= Significant (P≤0.05) NS= Non-significant (P≥0.05) | | | | |

**Mean square values for total cholesterol (TC) of rats**

| **Source** | **DF** | **SS** | **MS** | **F-Value** |
| --- | --- | --- | --- | --- |
| **Group** | 4 | 1619.21 | 404.803 | 149.08** |
| **Days** | 3 | 20.63 | 6.878 | 2.53 ^NS^ |
| **Group*Days** | 12 | 1059.73 | 88.311 | 32.52** |
| **Error** | 40 | 108.61 | 2.715 |  |
| **Total** | 59 | 2808.19 |  |  |
| **= Highly significant (P≤0.01) *= Significant (P≤0.05) NS= Non-significant (P≥0.05) | | | | |

**Mean square values for triglyceride (TG) of rats**

| **Source** | **DF** | **SS** | **MS** | **F-Value** |
| --- | --- | --- | --- | --- |
| **Group** | 4 | 122.72 | 30.68 | 13.94** |
| **Days** | 3 | 69.73 | 23.24 | 10.56** |
| **Group*Days** | 12 | 404.23 | 33.69 | 15.31** |
| **Error** | 40 | 88.02 | 2.201 |  |
| **Total** | 59 | 684.71 |  |  |
| **= Highly significant (P≤0.01) *= Significant (P≤0.05) NS= Non-significant (P≥0.05) | | | | |

**Mean square values for low-density lipoproteins (LDL) of rats**

| **Source** | **DF** | **SS** | **MS** | **F-Value** |
| --- | --- | --- | --- | --- |
| **Group** | 4 | 1363.46 | 340.87 | 148.03** |
| **Days** | 3 | 65.57 | 21.86 | 9.49** |
| **Group*Days** | 12 | 344.75 | 28.72 | 12.48** |
| **Error** | 40 | 92.11 | 2.30 |  |
| **Total** | 59 | 1865.89 |  |  |
| **= Highly significant (P≤0.01) *= Significant (P≤0.05) NS= Non-significant (P≥0.05) | | | | |

**Mean square values for high-density lipoproteins (HDL) of rats**

| **Source** | **DF** | **SS** | **MS** | **F-Value** |
| --- | --- | --- | --- | --- |
| **Group** | 4 | 455.07 | 113.769 | 49.07** |
| **Days** | 3 | 88.54 | 29.514 | 12.73** |
| **Group*Days** | 12 | 260.78 | 21.732 | 9.37** |
| **Error** | 40 | 92.74 | 2.318 |  |
| **Total** | 59 | 897.14 |  |  |
| **= Highly significant (P≤0.01) *= Significant (P≤0.05) NS= Non-significant (P≥0.05) | | | | |

**Mean square values for total antioxidant capacity (TAC) of rats**

| **Source** | **DF** | **SS** | **MS** | **F-Value** |
| --- | --- | --- | --- | --- |
| **Group** | 4 | 3.222 | 0.805402 | 309.18** |
| **Days** | 3 | 2.878 | 0.959415 | 368.30** |
| **Group*Days** | 12 | 1.610 | 0.134152 | 51.50** |
| **Error** | 40 | 0.1042 | 0.0026005 |  |
| **Total** | 59 | 7.8139 |  |  |
| **= Highly significant (P≤0.01) *= Significant (P≤0.05) NS= Non-significant (P≥0.05) | | | | |

**Mean square values for total oxidative stress (TOS) of rats**

| **Source** | **DF** | **SS** | **MS** | **F-Value** |
| --- | --- | --- | --- | --- |
| **Group** | 4 | 13.7320 | 3.43300 | 154.26** |
| **Days** | 3 | 9.5558 | 3.18525 | 143.13** |
| **Group*Days** | 12 | 9.5918 | 0.79932 | 35.92** |
| **Error** | 40 | 0.8902 | 0.02226 |  |
| **Total** | 59 | 33.7698 |  |  |
| **= Highly significant (P≤0.01) *= Significant (P≤0.05) NS= Non-significant (P≥0.05) | | | | |
